# Streamlined and on-demand preparation of mRNA products on a universal integrated platform

**DOI:** 10.1038/s41378-023-00538-8

**Published:** 2023-07-24

**Authors:** Hongjuan Wei, Zhen Rong, Liyan Liu, Ye Sang, Jing Yang, Shengqi Wang

**Affiliations:** Bioinformatics Center of AMMS, Beijing, 100850 P. R. China

**Keywords:** Nanoparticles, Materials science

## Abstract

Vaccines are used to protect human beings from various diseases. mRNA vaccines simplify the development process and reduce the production cost of conventional vaccines, making it possible to respond rapidly to acute and severe diseases, such as coronavirus disease 2019. In this study, a universal integrated platform for the streamlined and on-demand preparation of mRNA products directly from DNA templates was established. Target DNA templates were amplified in vitro by a polymerase chain reaction module and transcribed into mRNA sequences, which were magnetically purified and encapsulated in lipid nanoparticles. As an initial example, enhanced green fluorescent protein (eGFP) was used to test the platform. The expression capacity and efficiency of the products were evaluated by transfecting them into HEK-293T cells. The batch production rate was estimated to be 200–300 μg of eGFP mRNA in 8 h. Furthermore, an mRNA vaccine encoding the receptor-binding domain (RBD) of the severe acute respiratory syndrome coronavirus 2 (SARS-CoV-2) spike protein was produced by this platform. The proposed integrated platform shows advantages for the universal and on-demand preparation of mRNA products, offering the potential to facilitate broad access to mRNA technology and enable the development of mRNA products, including the rapid supply of new mRNA-based vaccines in pandemic situations and personalized mRNA-based therapies for oncology and chronic infectious diseases, such as viral hepatitis and acquired immune deficiency syndrome.

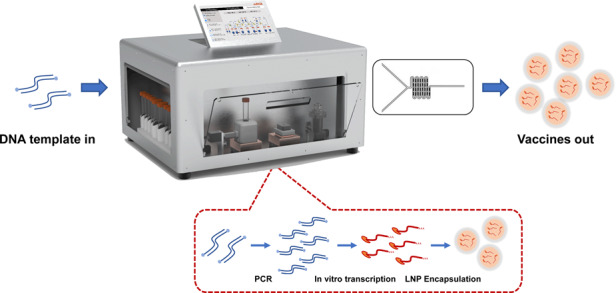

## Introduction

Since its outbreak in December 2019, coronavirus disease 2019 (COVID-19) has caused immense losses worldwide^1^. Mass vaccination campaigns are regarded as the key to containing the pandemic and reducing the mortality rate. Great efforts have been made toward the development of prophylactic vaccines, including inactivated vaccines, recombinant subunit vaccines, adenovirus vector vaccines, and mRNA vaccines. Several vaccines have already entered large-scale use and have received real-world feedback data^2–7^. As of 11 October 2022, a total of 12,782,955,639 vaccine doses have been administered globally (https://covid19.who.int/). The manufacturing process for conventional vaccines must be tailored to each specific disease because of the varied production and purification process for different pathogens. Thus, developing a safe and effective vaccine for an emerging infectious disease, such as COVID-19, is relatively expensive, complicated, and time-consuming, thus making it difficult to rapidly respond to an urgent epidemic^8,9^. However, the emergence of nucleic acid vaccines in recent years has provided revolutionary ideas for these circumstances^10^. Nucleic acid vaccines take a shorter time from development to production than conventional vaccines and are thus more appropriate for effective “on-demand” epidemic and pandemic responses^11^.

Several DNA or mRNA vaccines have been administered either by direct injection or through delivery systems for prophylactic and therapeutic applications in cases of infectious disease or cancer^12,13^. However, DNA vaccines are generally considered less safe due to the potential risk of mutagenesis^14^. In contrast, mRNA can be translated into proteins in the cytoplasm with no risk of gene insertion^15^. In addition, mRNA is known to take part in the majority of biochemical processes in the human body, transmitting genetic information to many proteins. Therefore, mRNA has the capacity to be developed into a versatile vaccine platform addressing viral, fungal, bacterial, and autoimmune diseases; allergies; and tumors^11,16–21^. In the past few decades, remarkable achievements have been made in the stability, biocompatibility, biosecurity, and delivery vectors of mRNA vaccines, which are now considered promising prophylactic vaccines for infectious diseases and therapeutic vaccines for cancer immunotherapy^22,23^. mRNA vaccines can induce stronger cellular and humoral responses because many epitopes are presented by class I and class II major histocompatibility complexes^24^. Moreover, the prospects of mRNA vaccines have been confirmed by the effective, long-lasting, and safe immune responses observed in animal models and the data from human clinical trials and the real world^25,26^. mRNA vaccines notably show great superiority in COVID-19 vaccine competition due to their advantages of rapid response and simple manufacturing^27–30^. In December 2020, the Food and Drug Administration of the USA issued emergency approvals for two mRNA vaccines against COVID-19 (Moderna [Cambridge, USA] and Pfizer [New York, USA]/BioNTech [Mainz, Germany])^31–33^. The mRNA-1273 vaccine from Moderna showed 94.1% efficacy at preventing COVID-19 illness, including severe cases^34^. The real-world trial in Israel revealed the effectiveness of two doses of BNT162b2 from Pfizer/BioNTech against a range of SARS-CoV-2 outcomes across all age groups^35^. The successful implementation of mRNA vaccines to address the COVID-19 pandemic has shown the tremendous potential of this technology.

mRNAs are currently produced through in vitro transcription (IVT), which is a versatile and mature technique^36,37^. This production process is independent of sequence, and it could be used to produce nearly any mRNA molecule of similar size, with relatively minor adjustments. According to public information, Pfizer/BioNTech produced their BNT162b2 vaccines starting from plasmids containing the genetic instructions for a human cell to build coronavirus proteins and trigger an immune response to the virus. After vials of DNA templates were harvested, strands of mRNA were transcribed, purified, and encapsulated into lipid nanoparticles (LNPs). Alternatively, mRNA could be generated from polymerase chain reaction (PCR)-amplified cDNA templates equipped with a bacteriophage RNA polymerase promoter located on the transcription start site. A cap analog must be added to the 5′ end of the mRNA, either cotranscriptionally or posttranscriptionally, by using a recombinant enzyme. DNA digestion with DNases is necessary to eliminate DNA contamination. A poly(A) tail is then added to the 3′ end of the mRNA by *E. coli* poly(A) polymerase to further stabilize the mRNA^38^. After purification and encapsulation with lipid vectors, the mRNA vaccines are ready to test for further optimization and application. During mRNA manufacturing, RNases should be carefully avoided because they could substantially reduce the yield of mRNA^9^. This manufacturing process could clearly be automated. Moreover, automated integrated mRNA preparation platforms could be powerful tools for mRNA research and development, with products such as mRNA vaccines and mRNA-based drugs. However, few reports about automated mRNA preparation platforms are available. A digital-to-biological converter (DBC) could produce biologics, such as DNA templates, RNA molecules, proteins, and viral particles, “on demand”, making it powerful for adaptation to many applications, but it is also bulky^39^. Automated microfluidic systems addressing the individual biochemical requirements of transcription and translation steps in separate compartments could also be developed^40^. However, the disadvantages of microfluidic devices are their limited reaction volumes and lack of versatility. Therefore, automated “on-demand” mRNA preparation platforms for quick responses to urgent epidemic issues and personalized treatments are still urgently needed.

Herein, a universal integrated platform with a corresponding control system for the streamlined and on-demand preparation of mRNA products is reported. The platform had three main components: (1) a PCR module to amplify the target DNA templates; (2) a heating–magnet separating–mixing (HMM) module to provide a mixing platform for thermostable, magnetically separable reaction components for IVT; and (3) an LNP module to directly encapsulate mRNA into LNPs by using staggered herringbone micromixer (SHM) microfluidic chips. Specifically, the automated production of mRNA was fulfilled through a series of workflow files customized by users in accordance with their specific needs. In this work, enhanced green fluorescent protein (eGFP) was taken as an initial example to develop a group of workflow files, including washing, self-testing, PCR amplification, IVT with capping, DNA digestion, poly(A) tailing, and purification. The integrated platform was then utilized to carry out PCR and IVT reactions for eGFP mRNA production and LNP encapsulation. Furthermore, a SARS-CoV-2 RBD-mRNA vaccine was produced by this platform. Western blotting analysis of SARS-CoV-2 RBD protein expression indicated the practicability of this platform. The proposed integrated platform shows advantages for the universal, flexible, and automated preparation of mRNA products and thus has the potential to facilitate broad access to mRNA technology and enable mRNA product development.

## Experimental section

### PCR

The PCR mix contained 10 U of LA Taq (Takara, Beijing, China), 10× LA Taq buffer II (Mg^2+^ Plus), dNTP Mixture (200 nM each), 4.8 ng DNA Template, Prime F and R (200 nM each), and nuclease-free water up to 200 μL. Primers were synthesized by Sangon Biotech (Shanghai, China). The parameters of the reaction were 95 °C for 5 min; 30 cycles of 95 °C for 40 s, 58 °C for 45 s, and 72 °C for 60 s; and a final extension of 72 °C for 10 min. DNA products were qualitatively detected by electrophoresis in a 1% agarose TAE gel. Afterward, large amounts of DNA were captured by hydrophilic-coated streptavidin magnetic beads (New England Biolabs, Ipswich, USA) in 1 mL buffer with NaCl (0.72 M), Tris-HCl (20.0 mM, pH 7.5), and EDTA (1.0 mM) at 30 °C for 45 min.

### IVT

The IVT reaction mix contained 20 µL of T7 polymerase enzyme mix (Promega, Madison, USA); 5× buffer; 1.875 mM each of rATP, rCTP, and rUTP; 0.225 mM rGTP, 0.375 mM cap analog (Promega, Madison, USA), linear DNA captured by hydrophilic-coated streptavidin magnetic beads from the last step, and nuclease-free water up to 200 µL. The mixture was incubated at 37 °C for 2 h. Then, 10 U of RQ1 RNase-free DNase enzyme (Promega, Madison, USA) was added to digest the DNA templates at 37 °C for another 15 min. Afterward, 200 μL of the mixture from the last step was incubated with 40 U of E-PAP (Fisher Scientific or Invitrogen, Gibco, USA), 5× E-PAP buffer, 2.5 mM MnCl_2_, 1 mM ATP, and nuclease-free water up to 400 μL at 37 °C for 1 h. Oligo d(T) 25-coated magnetic beads (New England Biolabs, Ipswich, USA) were used to purify the mRNA products through specific binding of oligo d(T) and the poly(A) tail. In brief, 100 μL of binding buffer containing 40.0 mM Tris-HCl (pH 7.5), 1.6 M LiCl, 3.2 μL of 2.0 mM EDTA, and 0.5 U/μL RNase inhibitor was used to wash 200 μL of oligo d(T) 25-coated magnetic beads once and resuspend the magnetic beads. After incubation for 5 min at 65 °C, the mRNA products were mixed immediately with magnetic beads for 5 min at room temperature. Next, the supernatant was discarded, and the magnetic beads were washed twice with 200 μL of washing buffer containing 10.0 mM Tris-HCl (pH 7.5), 0.15 M LiCl, 1 mM EDTA, and 0.5 U/μL RNase inhibitor. Finally, 200 μL of nuclease-free water was used to elute the purified mRNA. The quality and concentration of the synthesized mRNA were authenticated using an Agilent 2100 Bioanalyzer and RNA Nano 6000 Assay Kit (Agilent). DEPC-treated water (0.1%) was used in all solution preparations unless specifically mentioned. All centrifuge tubes were autoclaved, and all surfaces were treated with RNase Zap (Fisher Scientific, Gibco, USA) and 0.1% DEPC-treated water to eliminate any possible RNase contamination.

### LNP encapsulation

The combined pressure controller (Flow EZ, 0–2000 mbar) and the flow monitoring module (FLOW UNIT, 0–1000 μL/min) from Fluigent (France) were used for constant flow control in the LNP encapsulation process. SHM microfluidic chips purchased from Ibiochips were adapted for the rapid mixing process. Images of the LNPs were taken via transmission electron microscopy (Hitachi H-7650, Japan). The dynamic light scattering (DLS) distributions of the LNPs were characterized using a Nano-Lab Zeta Sizer (Malvern, UK)^41,42^.

### In vitro transfection

HEK-293T (ATCC, CRL-3216) cells maintained in Dulbecco’s modified Eagle’s medium (Thermo Fisher Scientific) with 10% fetal bovine serum (Thermo Fisher Scientific) and 1% antibiotics (penicillin 100 U/mL penicillin–100 μg/mL streptomycin, Thermo Fisher Scientific) were transfected with mRNA-LNP complexes to further test the quality of the transcribed mRNA. One day before transfection, the cells were seeded in a 12-well plate at a density of 3 × 10^5^ cells per well. The mRNA-LNP complexes were added to the cells (1 μg/well). Protein expression was detected at 24 h post-transfection. When eGFP mRNA was applied, green fluorescent protein expression was observed under an Olympus IX71 fluorescence microscope, and images were obtained by DP controller software. Alternatively, when SARS-CoV-2-mRNA was applied, SARS-CoV-2 RBD protein expression was analyzed by Western blotting. The mRNA-LNP produced by the commercial Precision Nanosystems Ignite (Vancouver, British Columbia, Canada) was used as a positive control.

## Results and discussion

### System design of the platform

**Fig. 1:**
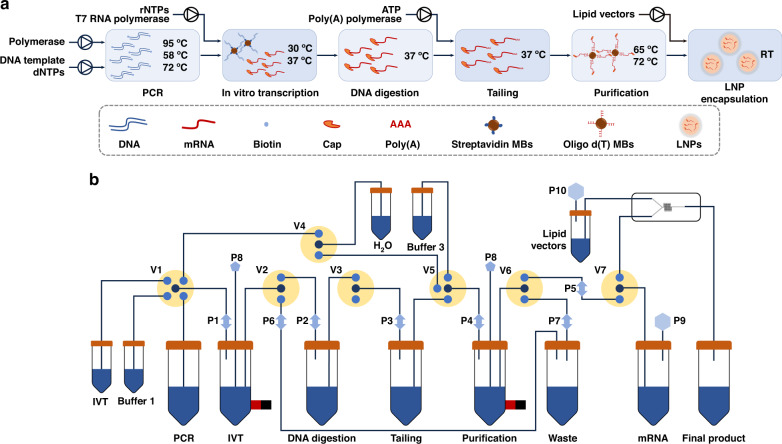
System design of the platform. **a** Schematic of the mRNA production process adapted in this system, including PCR, IVT, digestion of the DNA template, poly(A) tailing, purification of mRNA transcripts, and LNP encapsulation. **b** Liquid flow diagram of the integrated “template-in vaccines-out” platform for mRNA preparation

An integrated “template-in vaccines-out” platform was designed for the convenient preparation of mRNA vaccines to meet the demands of automated “on-demand” mRNA manufacturing devices for quick responses to urgent epidemic issues and personalized treatments. The mRNA production process adapted in this system included PCR, IVT, digestion of DNA templates, poly(A) tailing, purification of mRNA transcripts, and LNP encapsulation, as shown in Fig. 1a. When the target vaccine was determined by sequencing or bioinformatics, the template could then be designed to contain. untranslated regions (UTRs) and the gene of interest (GOI). After several cycles of PCR amplification, large amounts of DNA templates were captured by hydrophilic-coated streptavidin magnetic beads. Then, the IVT reaction mix was introduced for several hours of IVT. Afterward, the DNA templates were degraded by DNase. Then, a ≥150 base poly(A) tail was added to the RNA transcripts through poly(A) polymerase. Next, the mRNA products were enriched by oligo d(T) 25-coated magnetic beads. After elution and LNP encapsulation were performed, the mRNA vaccines were ready to use. Figure 1b illustrates the liquid flow diagram of the integrated “template-in vaccines-out” platform for mRNA preparation. All reagents were separately loaded, and thus, cross-contamination was avoided during the whole experiment. After the experiment, the used storage units were disposed, and all reagents were kept within the waste reservoirs.

### Structure of the platform

**Fig. 2:**
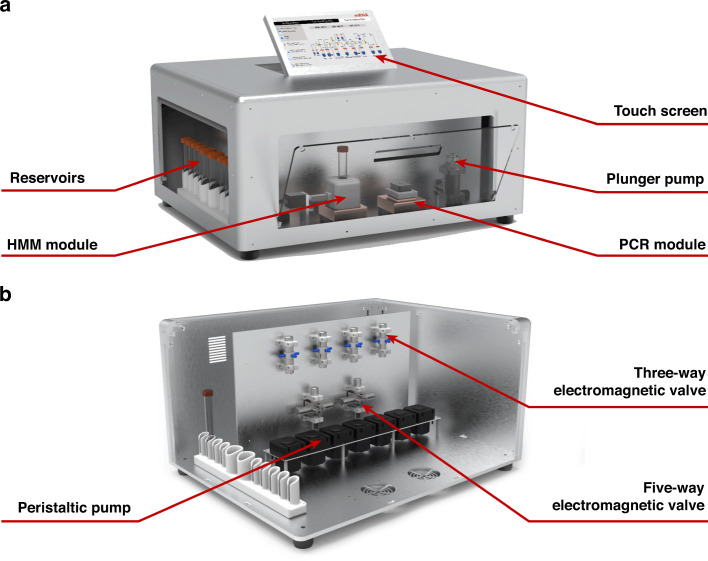
Structure of the prototype. **a** Overall view of the prototype, mainly including the PCR module, HMM module, plunger pump, and reservoirs. **b** Structures inside the prototype, mainly including seven peristaltic pumps, two five-way electromagnetic valves, and four three-way electromagnetic valves

An integrated system was designed to realize automated PCR and IVT for mRNA preparation. The prototype was designed using SolidWorks 3D software (Concord, USA), as shown in Fig. 2a. The main material used in this prototype was aluminum alloy, with some 3D-printed attachments. The prototype was 62.5 cm × 50.0 cm × 32.5 cm. It contained four areas, as shown in Fig. 2b. The back area was designed for electronic parts, including several circuits and a power supply. The middle area was filled with seven peristaltic pumps, two five-way electromagnetic valves, and four three-way electromagnetic valves joined together by tubing (FEP, 1/16′′). The left side contained 11 reservoirs (nine reagent reservoirs, one washing reservoir for the plunger pump, and another reservoir for unwanted waste). The front area was used for PCR and IVT reactions, and a plunger pump was used to mix the reaction reagents. The left and front areas were designed with a door made of polymethyl methacrylate, which enables user-friendly implementation of the PCR and IVT steps. In addition, a microcomputer with a touch screen was placed on top of the whole device, through which users could customize their workflows and conduct experiments. The key components of the platform are the PCR module and HMM module for PCR and IVT, respectively, which were both connected to three control circuits and a power-supply circuit, as shown in Fig. S1.

The platform used customized user-specific workflow files composed of a series of instructions for components such as valves to control the whole reaction. The introduction of workflows increased the flexibility, versatility, and transportability of the platform. In this work, eGFP was chosen as an example to develop a set of workflow files on the basis of experimental details mentioned earlier in the Materials and Methods. Figure S2 shows the user-friendly interface for workflow editing. These workflow files were then sequentially loaded to the platform, and the automated reaction took place with a few manual interventions, such as adding liquid mix and changing reaction tubes. Before a new round of mRNA was prepared, a “test” file was loaded for the prototype’s self-test, and the tubing was rinsed in accordance with a “wash” file: once with RNase ZAP and then three times with DEPC-treated water (0.1%) to remove carryovers from previous experiments and any possible RNases. During the performance of workflows, the real-time temperature was shown on the top of the scheme, and the executed flows turned gray, as shown in Figure S3. Throughout the preparation process, the users needed to load reaction reagents and buffers into the corresponding reservoirs and then choose a workflow file and press the “run” button. Notably, this platform is universal and could be used to produce several other mRNA products. The workflow files used in this article are detailed in the Supporting Information.

### PCR module

**Fig. 3:**
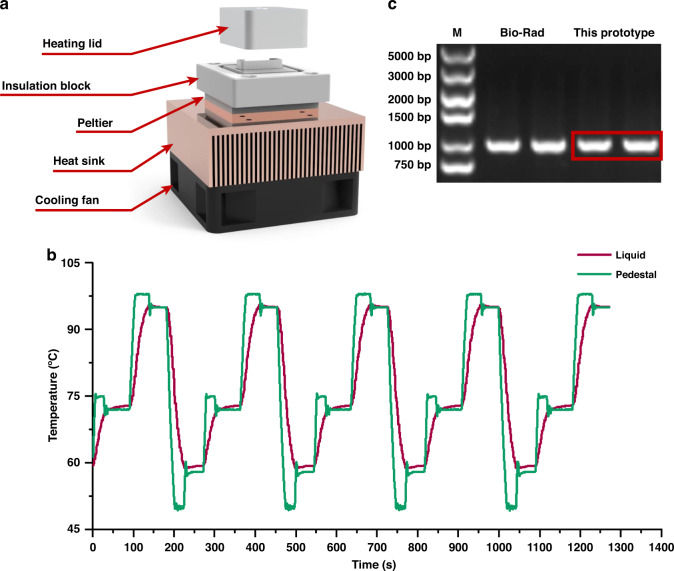
PCR module. **a** Structure of the PCR module, including a cooling fan, a heat sink, a Peltier thermoelectric cooling effect element, an insulation block, a reaction tube, and a heating lid. **b** Real-time temperature curves in PCR (red for liquid and green for tube pedestal). **c** Electrophoresis image after PCR, where samples were loaded in the following order: marker, PCR products from the standard machine, and PCR products from this platform

One of the key components of the platform is the PCR module, as shown in Fig. 3a. The PCR module consists of a supportive base composed of a cooling fan, copper alloy heat sink, Peltier thermoelectric cooling effect element, PT-1000 temperature sensor, custom designed insulation block, aluminum alloy tube pedestal, and heating lid. This simple module could achieve fast temperature change, with a maximum heating rate of 1.8 °C/s (72–95 °C) and a cooling rate of 2.6 °C/s (95–58 °C) for the reagents. The PCR temperature control strategy adopted a “pedestal-first liquid-next” strategy by setting the tube pedestal to a higher or lower temperature to change the liquid temperature quickly. When the temperature of the reaction liquid almost reached the target temperature, the tube pedestal’s temperature was restored to the target temperature and maintained for PCR, as shown in Fig. 3b. After PCR amplification, the DNA products were quantitatively evaluated by electrophoresis in a 1% agarose TAE gel. As shown in Fig. 3c, the DNA products from the PCR module exhibited similar sizes and amounts to those from the commercialized PCR machine (Bio-Rad T100 Thermal Cycler).

### HMM module

**Fig. 4:**
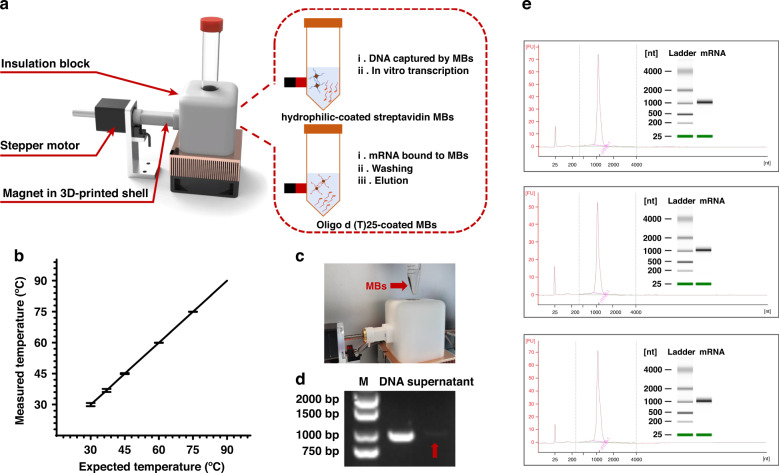
HMM module. **a** Structure of the HMM module, including a cooling fan, a heat sink, a Peltier thermoelectric cooling effect element, an insulation block, and a magnet contained in a 3D-printed shell controlled by a stand-alone stepper motor. **b** Heating stability of the IVT module (a maximum temperature fluctuation of ±0.5 at 30 °C). **c** Picture of magnetic beads gathered together by a magnet wrapped in a 3D-printed shell (in less than 90 s). **d** Electrophoresis image after DNA templates were captured by hydrophilic-coated streptavidin magnetic beads, where samples were loaded in the following order: marker, PCR products, and supernatant after magnetic bead capture. **e** Three batches of mRNA synthesized and purified in vitro were quantified by the Agilent 2100 bioanalyzer system by comparison with the heat-denatured Agilent RNA 6000 ladder

The other key component of the platform is the HMM module for IVT, as shown in Fig. 4a. The HMM module also had a supportive base composed of a cooling fan, a copper alloy heat sink, a Peltier thermoelectric cooling effect element, a PT-1000 temperature sensor, another specially designed insulation block, and an aluminum alloy tube pedestal. The insulation block wrapped in the periphery could ensure the stability of the reaction temperature. The HMM module could provide a stable temperature environment for IVT, with a maximum temperature fluctuation of ±0.5 °C at 30 °C (Fig. 4b). A stand-alone stepper motor controlled the movement of the magnet contained in a 3D-printed shell. The HMM module employed a plunger pump to mix the reaction containing magnetic beads, with optimized parameters consisting of steps, speed, interval time, and loops. Then, large numbers of DNA templates were captured on hydrophilic-coated streptavidin magnetic beads, and later, mRNA products were captured on oligo d(T) 25-coated magnetic beads. A traditional oscillating cultivator mixes liquid in a constant shaking reaction tube, which requires cumbersome external hardware. The proposed platform adopted a plunger pump instead, further decreasing the size of the whole prototype. Moreover, the magnetic beads in the reaction mix could be gathered by a magnet wrapped in a 3D-printed shell in less than 90 s (Fig. 4c). The electrophoresis image (Fig. 4d) showed no obvious band of supernatant, indicating the practicability of the HMM module. After the DNA templates were captured, the IVT reaction mix was introduced into streptavidin magnetic beads, followed by digestion of DNA, poly(A) tail modification of the transcripts, and purification. mRNAs with the desired size were synthesized and purified in vitro and then quantified by an Agilent 2100 bioanalyzer system. Figure 4e shows three different batches of mRNAs synthesized and purified in vitro in comparison with the heat-denatured Agilent RNA 6000 ladder, with yields of 308 (1540 ng/μL), 198 (990 ng/μL), and 324 (1620 ng/μL) μg. For reference, 100 μg of Moderna mRNA-1273 for COVID-19 was used in phase 3 randomized, observer-blinded, placebo-controlled trial^34^. In addition, 30 μg of BNT162b2 from Pfizer/BioNTech was used in phase 3 clinical trial to evaluate safety and efficacy^43^. Therefore, the yield of the platform was sufficient for three independent experiments validating the candidate mRNA vaccine, which can further speed up the process for new mRNA products from the laboratory to the clinic.

### LNP module and in vitro transfection

**Fig. 5:**
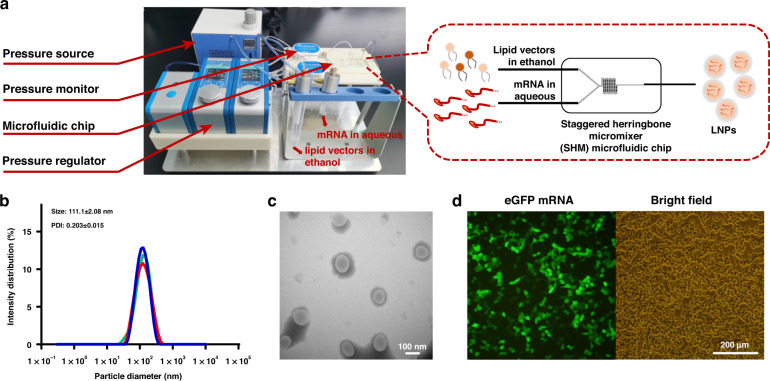
LNP module and in vitro transfection. **a** Image of the LNP encapsulation device using microfluidic chips, including a pressure source, two monitoring modules, and two pressure controllers for mRNA in water and lipid vectors in ethanol. **b** Size distribution of LNPs determined by DLS. **c** Morphology of LNPs by TEM. Scale bar, 100 nm. **d** LNP transfection in 293T cells imaged by fluorescence microscopy. Scale bar, 200 μm

A pressure controller, flow monitoring module, and SHM microfluidic chips (Fig. 5a) were set up in combination to generate LNPs^21,44^. The LNPs were formed by electrostatic interaction when the lipid vectors dissolved in ethanol mixed with a nucleic acid solution at a velocity rate of 1:3 (300 μL/min for lipid vectors and 900 μL/min for mRNA vaccine). The hydrodynamic diameter of the LNPs generated was analyzed by DLS, which revealed an average size of 111.1 nm (Fig. 5b). The morphology of the LNPs generated was determined by TEM. Figure 5c illustrates that the LNPS were spherical in shape, and the size was consistent with the results of DLS. These results confirmed the ability to form stable LNPs of lipid vectors and mRNA. Following automated preparation and encapsulation on the platform, the quality of the products was further evaluated by transfection into HEK-293T cells. Figure 5d shows that eGFP was successfully expressed in the cytoplasm, with bright green fluorescence in positive cells under the fluorescence microscope. In summary, these results indicated the practical application of the system for mRNA vaccine preparation.

### Application in SARS-CoV-2 RBD-mRNA vaccine production

**Fig. 6:**
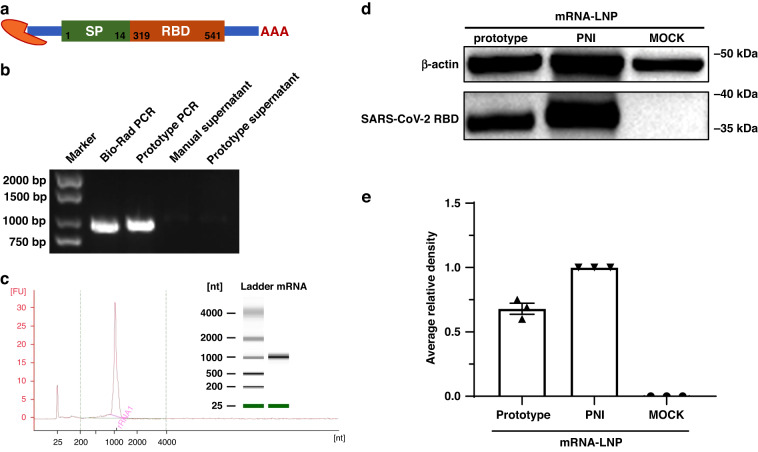
Application in SARS-CoV-2 RBD-mRNA vaccine production. **a** A SARS-CoV-2 RBD-mRNA vaccine was constructed from optimized codons encoding the signal peptide (SP, 1–14 residues) and the receptor-binding domain (RBD, 319–541 residues) of the SARS-CoV-2 spike protein. **b** Electrophoresis image of RBD, where samples were loaded in the following order: marker, PCR products from the standard machine, PCR products from this platform, supernatant after magnetic bead capture by manual operation, and supernatant after magnetic bead capture by the platform. **c** RBD-mRNA synthesized and purified in vitro with the desired size quantified by an Agilent 2100 bioanalyzer system. **d** Western blotting analysis and (E) average relative density of SARS-CoV-2 RBD protein expression from mRNA-LNP produced on the proposed platform and PNI-prepared mRNA-LNP at 24 h post-transfection. PNI stands for Precision Nanosystems Ignite. The average relative density was assessed by gray analysis using ImageJ software

After the function of the mRNA produced by this platform with the established workflow files was tested with eGFP expression, the SARS-CoV-2 RBD-mRNA vaccine developed by the authors’ group was used to further test this platform^42^. As shown in Fig. 6a, the coding sequence region starting with AUG encoded the signal sequence (residues 1–14) and RBD (residues 319–541) of the spike glycoprotein gene of the SARS-CoV-2 Wuhan-Hu-1 isolate (GenBank accession number, NC_045512)^42^. Figure 6b shows the electrophoresis image of RBD templates amplified by a standard machine or the PCR module in the proposed platform and their corresponding supernatants after purification by manual operation or by an HMM device. RBD-mRNA with the desired size was synthesized and purified in vitro, then quantified by an Agilent 2100 bioanalyzer system, as shown in Fig. 6c, with a yield of 280 μg (1400 ng/μL). The mRNA-LNP produced by the commercial Precision Nanosystems Ignite (PNI) was used as a standard control. As shown in Fig. 6d, we evaluated the protein expression levels of the mRNA-LNP prepared on the proposed platform and by PNI. Based on the relative density of the Western blot bands, the expression level of the mRNA-LNP prepared on the proposed platform was ~70% of that of the commercial instrument, as shown in Fig. 6e. This indicated a reasonable protein expression capability of the mRNA-LNP prepared by the proposed platform. Our further work will focus on improving the efficiency of this platform.

## Conclusions

In this work, a universal integrated platform with a corresponding control system for the streamlined and on-demand preparation of mRNA products was proposed. eGFP was first used as an initial example to develop a group of workflow files, including washing, self-testing, PCR amplification, IVT with capping, DNA digestion, poly(A) tailing, and purification. The integrated platform was then utilized to carry out PCR and IVT for the production and LNP encapsulation of eGFP mRNA. Furthermore, a SARS-CoV-2 RBD-mRNA vaccine was produced by this platform, and successful protein expression indicated the practicability of this system. These results indicated that this integrated and automated platform could be readily adapted for the rapid preparation of mRNA products with varied sequences, which may help to facilitate broad access to mRNA technology and enable mRNA product development. For example, candidate mRNA vaccines for urgent epidemic issues and personalized treatments could be rapidly generated and then tested in a short time at the early research and development stage. Moreover, the proposed platform could serve as a manufacturing end-to-end solution to allow application in resource-limited areas to further accelerate the progress of new mRNA products from the laboratory to the clinic.

## Supplementary information

Supplemental Material
