# Supplementary material for: Streamlined and on-demand preparation of mRNA products on a universal integrated platform
Source: Microsyst Nanoeng. 2023 Jul 24;9:97. doi: 10.1038/s41378-023-00538-8 (PMC10363538; doi:10.1038/s41378-023-00538-8)
Supplement: Supplementary file 1 — Supplemental Material [file 41378_2023_538_MOESM1_ESM.docx]

**Supplementary materials**

**Streamlined and on-demand preparation of mRNA products on a universal integrated platform**

*Hongjuan Wei†, Zhen Rong†, Liyan Liu, Ye Sang, Jing Yang*, Shengqi Wang**

Bioinformatics Center of AMMS, Beijing 100850, P. R. China.

†These authors contributed equally to this work.

**Corresponding author E-mail:**

*S.Q. Wang: sqwang@bmi.ac.cn

*J. Yang: jingyang0511@sina.com

1. **Control system**

The control system is designed to give specific instructions to all components of the prototype conveniently and efficiently. An electromagnetic valve-control circuit was used to control the status “on/off” of electromagnetic valves, with 16 ports occupied (Figure S1A). An integrated temperature-control circuit was employed including two ways of heating-cooling for PCR and IVT respectively and one way of heating for the PCR lid. Both excepted temperature and measured temperature were recorded in curves and numbers with a precision of 0.1 ℃ (Figure S1B). In addition, three motor-control circuits were used to control peristaltic pumps, plunger pump, and magnet-assembly. Motor-control is referred to the mode (relative or absolute), direction (forward or backward), state (operate, stop or set to zero), step number, and other parameters as operating speed, accelerated speed, working current and etc. (Figure S1C). Figure S2 shows the interface of editing a workflow, which is user-friendly. During the performing of workflows, real-time temperature is show on the top of the scheme and the executed flows turned gray, as is shown in Figure S3.


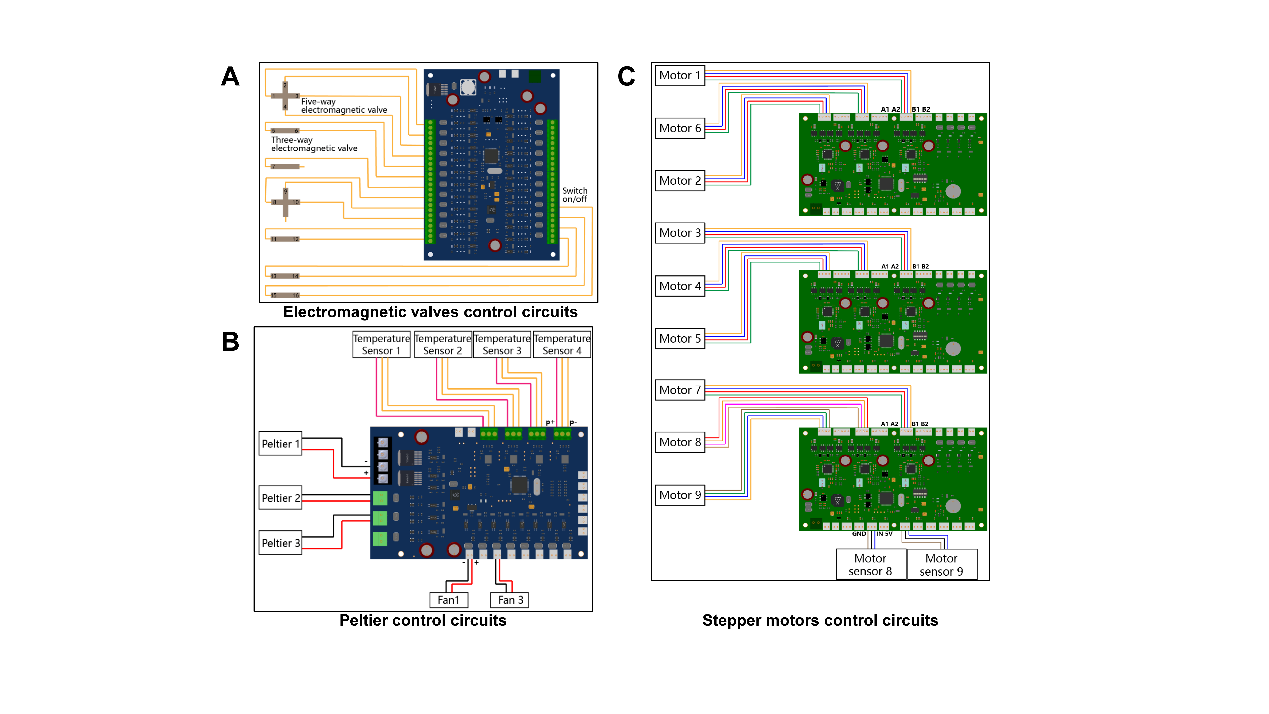


Figure S1. Diagram of electromagnetic valves (A), Peltier (B) and stepper motors (C) connecting to the circuits.


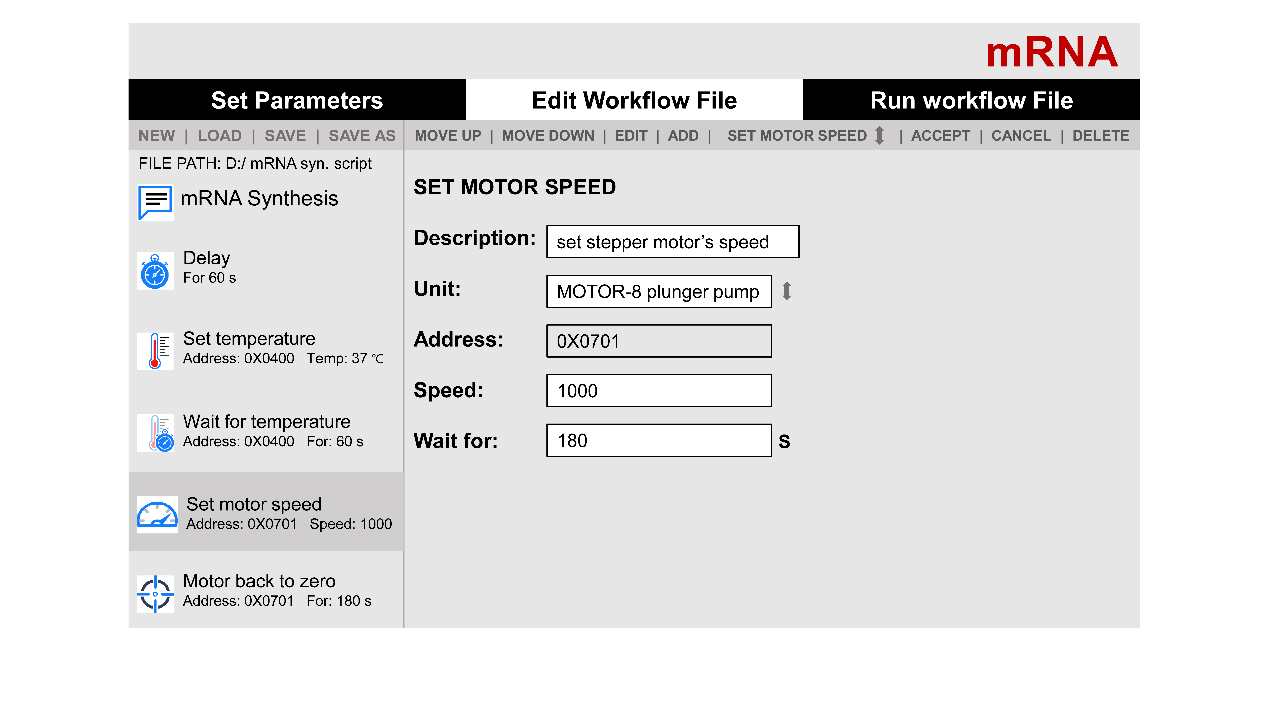


Figure S2. Image of the USER INTERFACE for workflow files editing.


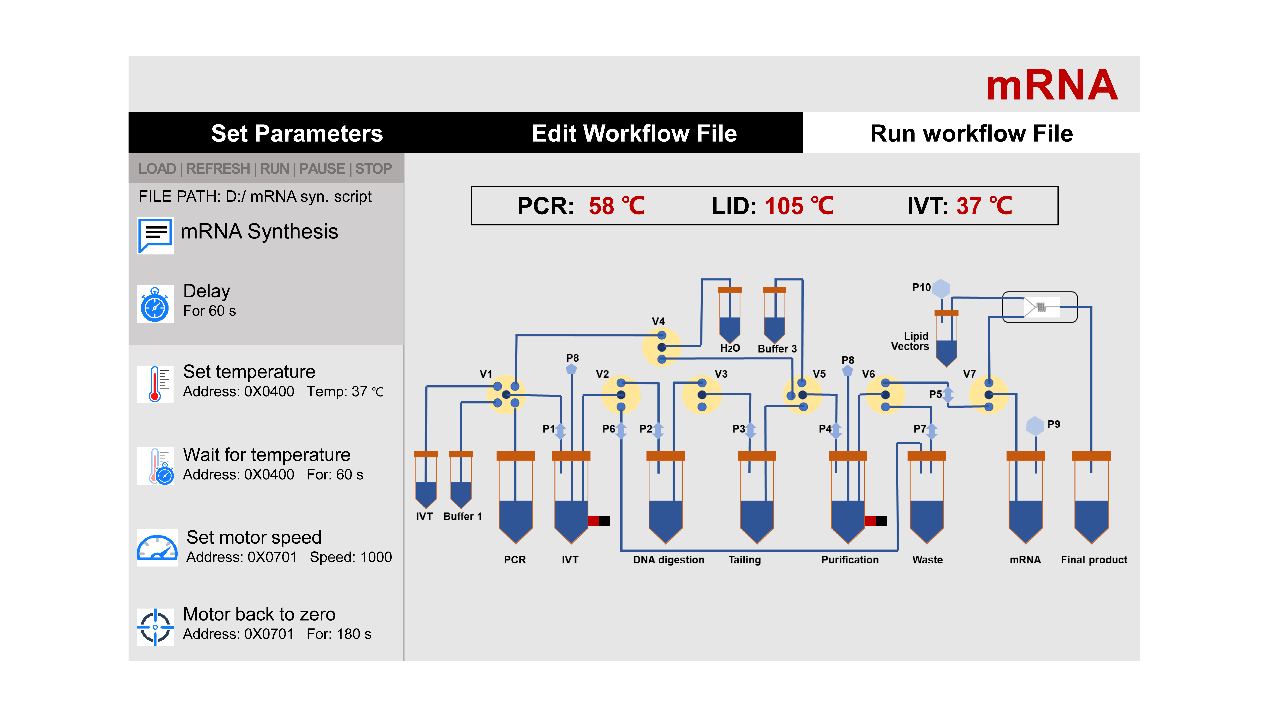


Figure S3. Image of the USER INTERFACE for workflow files running.

1. **Workflow files**

Customized workflows in this work mainly referred certain combination of these followed items: comment, delay, setting temperature, waiting for temperature, setting motor speed, setting motor to zero, moving motor, waiting for motor, controlling valve, starting loop, ending loop. Some of them is single-used as delay, while others in pairs such as starting loop and ending loop. A set of workflow files used in this work are listed briefly as follows:

**Washing:** this file is optional to get rid of carryovers from previous experiments and any possible RNases. During washing, each pump and valve is under sufficient cleaning. In this work, the washing file ran four times altogether, one with RNase ZAP and three with 0.1% DEPC-treated water.

**Self-testing:** a “test” file was loaded to examine the function of electromagnetic valves, motors, Peltiers, fans and etc. before each experiment.

**Manufacture:** as is mentioned in methods, several combinations of valves, pumps, and Peltier were employed to realize the automated reaction.
